# Right Ventricular Hypertrophy in Spontaneously Hypertensive Rats (SHR/NHsd) Is Associated with Inter-Individual Variations of the Pulmonary Endothelin System

**DOI:** 10.3390/biology13100752

**Published:** 2024-09-24

**Authors:** Alicia Langer, Rolf Schreckenberg, Klaus-Dieter Schlüter

**Affiliations:** Physiologisches Institut, Justus-Liebig University, Aulweg 129, D-35392 Giessen, Germany; alicia.v.langer@med.uni-giessen.de (A.L.); rolf.schreckenberg@physiologie.med.uni-giessen.de (R.S.)

**Keywords:** pulmonary hypertension, endothelin receptor type B, right heart failure

## Abstract

**Simple Summary:**

Hypertension is a main risk factor for heart disease, leading to ventricular hypertrophy and heart failure. Spontaneously hypertensive rats (SHRs) are a commonly used model to study the processes of hypertension and left ventricular hypertrophy. However, from the literature, it is unclear whether SHRs develop right ventricular hypertrophy or not. Here, seventy-six SHRs of the strain SHR/NHsd were analyzed and it was found that approximately 7% of them develop right ventricular hypertrophy in an age-independent way, indicating sub-strain-specific differences in SHR/NHsd. This study identified that SHRs with right ventricular hypertrophy differ from those without right ventricular hypertrophy by exhibiting low expression of the endothelin clearance receptor (endothelin receptor type B) in the lung, leading to increased local appearance of endothelin-1, a strong vasoconstrictor. In line with this observation, increased expression of α-smooth muscle actin further suggests that pulmonary hypertension triggers right ventricular hypertrophy in this sub-strain. Interestingly, in humans, right ventricular hypertrophy is also found in some but not all patients. Nevertheless, independent of severe right ventricular hypertrophy, the phenotype is mild and can be tolerated by the animals. In conclusion, our study helps to understand heterogeneous findings in SHRs in the literature.

**Abstract:**

Spontaneously hypertensive rats (SHRs) develop severe hypertension and subsequently left ventricular hypertrophy. Whether they also develop right ventricular hypertrophy is not clear. We analyzed 76 female SHRs (strain SHR/NHsd) and observed severe right ventricular hypertrophy in 7% of these rats (SHR-RVH). Right ventricular hypertrophy did not correlate with the age of the rats and was already seen in one rat at the pre-hypertensive state. The current study investigated the molecular fingerprint of the lung and right ventricle from SHR-RVH and compared this first to SHRs that did develop left but not right ventricular hypertrophy, and second to normotensive rats without hypertrophy. Rats with right ventricular hypertrophy had a decreased expression of the endothelin-B receptor (*EDNRB*) in the lung, together with an increased protein content of endothelin-1 and an increased expression of *ACTA2A*. Furthermore, in the right ventricle, a down-regulation of the endothelin-A receptor (*EDNRA*) was found, consistent with a mild phenotype. The data suggest that in a sub-group of SHR/NHsd rats, low expression of the endothelin clearance receptor (endothelin-B receptor) in the lung triggers an increase in vascular resistance to the right ventricle that then triggers hypertrophy. Our study is the first description of a genetic variant in a defined SHR strain.

## 1. Introduction

Spontaneously hypertensive rats (SHRs) are widely used as a model of essential hypertension. These animals develop hypertension from week eight on. Hypertension lasts life-long in these rats. Sub-strains, such as SHR-SP (spontaneously hypertensive rats—stroke prone), have even more severe hypertension and hypertrophy [1]. However, neither SHRs nor SHR-SPs normally develop right ventricular hypertrophy (RVH) [2,3]. There are few reports measuring pulmonary arterial pressure in SHRs, and collectively, these studies show that SHRs are more prone to develop pulmonary hypertension and subsequently RVH compared to normotensive rats [4,5,6]. However, most studies showed mild or no pulmonary hypertension in SHR in the co-presence of severe systemic hypertension [3,4,5].

In our own studies, we used female SHRs from the strain SHR-NHsd. Our own experiments were aimed at analyzing the effect of voluntary running wheel activity on hypertension [7,8,9,10]. In these studies, rats were sacrificed at the age of 2.5 months (pre-hypertensive stage), 7.5 months or 11.0 months (hypertensive stage). In most of these rats, right ventricular (RV) weights could not be distinguished from age- and sex-matched normotensive control rats. These findings were in accordance with the literature (as mentioned above). However, some rats displayed severe RVH intermittently. We defined the term severe RVH as RV weights that exceeded those of age- and sex-matched normotensive or hypertensive rats by more than two standard deviations. In general, RVH may translate into right heart failure after a short compensatory phase [11]. However, the SHR-NHsds with severe RVH identified in our studies were inconspicuous during normal housing.

Our observation, that SHR-NHsds develop RVH in some but not all cases, leads to two important questions: What triggers RVH in these rats? Why do they not develop severe right heart failure? To address these questions, we screened tissue samples for transcriptional alterations in the lung and right ventricle. The identification of differential regulated genes may help to understand mechanisms that drive the progression of pulmonary arterial hypertension and RVH.

## 2. Materials and Methods

### 2.1. Animal Model

This investigation is in agreement with the “Guide for the Care and Use of Laboratory Animals” published by the US National Institute of Health (NIH Publication No. 85-23, revised 1996). This study was approved by the local authorities (RP Gießen; V54-19 c 20 15 h 01 GI 20/1 Nr. 76/2014 and GI 20/1 Nr. 77/2014).

The tissues analyzed in this study were obtained from rats characterized before in detail and used for several projects [7,8,9,10]. To finalize all experiments, the rats were anesthetized by isoflurane inhalation. After cervical dislocation, the hearts were dissected from the lung, isolated and perfused via the Langendorff technique to remove blood contamination. Subsequently, all hearts were weighted, the atria were cut off and the free RV wall was separated. The weight of the free RV wall was considered as RV weight and the left ventricle, including the septum, as left ventricular weight. All organ weights were normalized to the tibia length of the animal. Subsequently, the tissues were quickly frozen in fluid nitrogen and stored until use at −80 °C.

### 2.2. Blood Pressure

The blood pressures of the rats analyzed in this study were reported before [9,12]. For technical details see [7,9]. All blood pressure measurements were performed by tail-cuff methods. In summary, the normotensive control rats that were included in this study had a systolic blood pressure of 125 ± 4 mmHg and a diastolic blood pressure of 83 ± 3 mmHg. The SHRs had a systolic blood pressure of 194 ± 11 mmHg and a diastolic blood pressure of 120 ± 13 mmHg. In the case of young pre-hypertensive rats, the Wistar controls had a systolic blood pressure of 131 ± 4 mmHg and a diastolic blood pressure of 79 ± 7 mmHg, while the SHRs had systolic and diastolic blood pressures of 128 ± 5 mmHg and 90 ± 1 mmHg, respectively.

### 2.3. RNA Isolation and Real-Time RT-PCR

Total RNA was isolated from lung and heart tissue using peqGold TriFast (peclab, Biotechnologie GmbH, Aschaffenburg, Germany) according to the manufacturer’s protocol. Genomic DNA contamination was avoided by use of 1 U DNase/µg RNA (Invitrogen, Karlsruhe, Germany). The synthesis of cDNA and subsequent Real-time PCR were performed as described before [7]. A list of primers used in this study can be found in the Supplement Table S1. For the screening of our samples, we used reference genes for several pathways. **Heart**: extracellular matrix (*TGFB1*, *BGN*, *DCN*, *COL1A1*, *COL3A1*, *CCN2*, *LOX*, *MMP12*), cardiac hypertrophy and differentiation (*NPPA*, *NPPB*, *MYH6*, *MYH7*, *ACTC1*, *ACTN2*, *FGF2*, *DES*, *DYN2*, *EFHD2*, *OSM*, *OSMR*, *CXCR4*, *CXCL12*, *GATA4*, *MEF2C*, *CCL2*, *CRYAB*, *MYO6*), cardiac function (*SLC8A1*, *ATP2A2*, *CASR*, *ADRB1*, *ADRB2*), oxidative stress (*SOD1*, *SOD2*, *SOD3*, *CYBA*, *CYBB*, *NFE2L2*), arginine metabolism (*ARG1*, *ARG2*, *NOS3*, *ODC1*, *NOS2*), mitochondrial function (*UCP2*, *UCP3*, *HADHA*, *PPARGC1A*), metabolism (*PCSK9*, *SST*, *SLC2A1*, *SLC2A4*), inflammation (*IL6*, *IL6R*), vascular biology (*CDH5*, *VEGFA*, *vWF*). **Lung**: extracellular matrix (*TGFB1*, *ELN*, *COL1A1*, *COL3A1*, *MMP2*, *MMP9*, *MMP12*), oxidative stress (*CYBA*, *CYBB*, *NCF1*, *NCF2*), lung development and function (*PTHLP*, *PTH1R*, *ODC1*, *PLIN2*), inflammation (*IL6*, *NOS2*, *PPARG*, *CXCR4*), vasotonus (*ADRB2*, *ADRB1*, *ACTA2*), endothelin system (*EDNRA*, *EDNRB*, *ECE1*, *EDN1*). Quantification is based on the 2^−ΔΔ^-method.

### 2.4. Western Blot

Protein extracts from tissues were generated as described before [7]. Primary antibodies were directed against endothelin-1 (anti-EDN1 antibody, Biozol, Eching, Germany; MBS2540132) and endothelin receptor type A (anti-ETAR antibody, BioSource, Kuiper, The Netherland; MBS9702013). Secondary antibodies (HRP-conjugated) directed against rabbit IgG were purchased from Affinity Biologicals (Ancaster, ON, Canada; P 0448).

### 2.5. ELISA

Plasma samples from rats were generated as previously described [9]. In these samples, we analyzed the amount of endothelin-1 with a commercial ELISA (Rat Endothelin-1 (ET-1) ELISA Kit, CSB-E06979r, Cologne, Germany). The detection limit of the ELISA was 2.5 pg/mL and the intra-assay variability was 3.9%.

### 2.6. Histology

Picro–Sirius red staining was used to visualize fibrosis, as described before [7]. Briefly, samples were embedded with Tissue-Tek^®^ (Sakura, Liden, The Netherlands) and sectioned in 10 µm slices. The slices were fixed with Bouin solution and subsequently stained in 0.1% (wt/vol) Sirius Red solution (Sigma-Aldrich Chemie, Taufkirchen, Germany). Sections were washed with 0.01 N HCl, Aqua dest. and dehydrated with ethanol.

### 2.7. Echocardiography

Echocardiography was performed as previously reported in detail [7]. Briefly, rats were anesthetized by isoflurane inhalation and the left ventricular function was assessed by two-dimensional echocardiography using a 12.5-MHz probe (Vivid I, GE Health Care, Chicago, IL, USA). Fractional shortening (FS) was calculated as left ventricular internal diameter in end-diastole (LVEDD) minus left ventricular internal diameter in end-systole (LVESD), divided by LVEDD and expressed as percentage (LVEDD-LVESD/LVEDD × 100).

### 2.8. Statistics

Normal distribution of data was validated by Shapiro–Wilk tests. Normal variance was subsequently analyzed using Levene’s test. Subsequently, two group comparisons were made using the *t*-test or the Mann–Whitney-U test where appropriate, to calculate the *p*-value. An ANOVA or a Kruskal–Wallis test with Student–Newman–Keuls or Bonferroni with correction for multiple testing was used for comparisons of more than two groups. In the figures, *p* < 0.05 is marked, and the exact *p*-value is indicated in the figure legend.

## 3. Results

### 3.1. Right Ventricular Hypertrophy in SHR/NHsd: Prevalence and Age-Dependency

**Figure 1:**
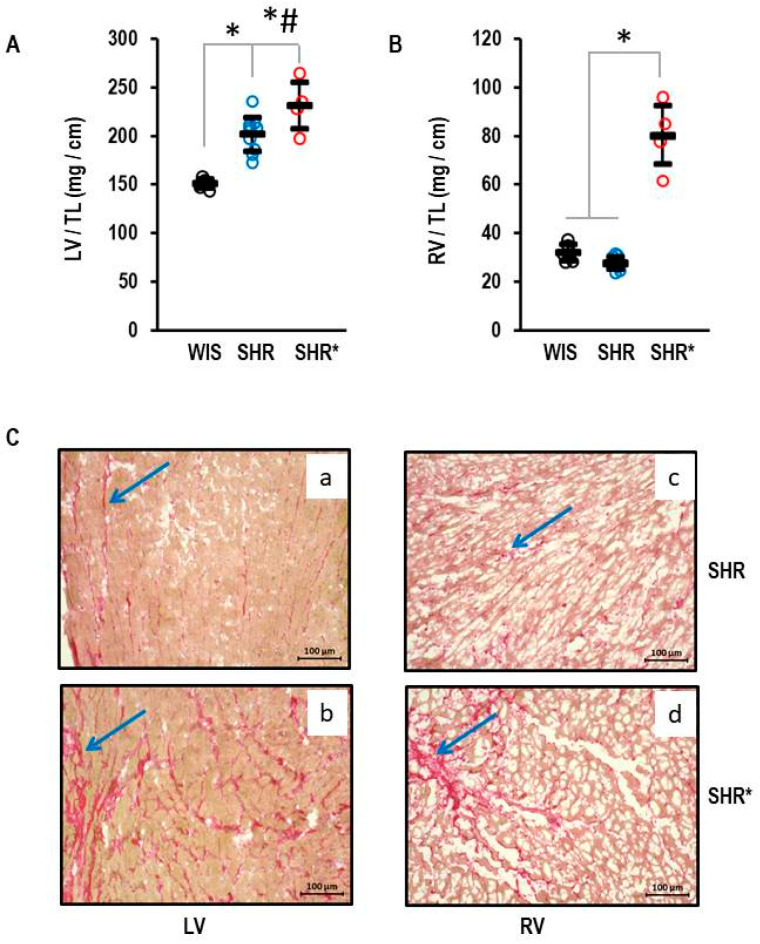
Heart weights of normotensive Wistar rats (WIS, *n* = 6), spontaneously hypertensive rats (SHR, *n* = 10), and spontaneously hypertensive rats with right ventricular hypertrophy (SHR*, *n* = 4). All ventricular weights are normalized to tibia lengths (TL). (**A**) Left ventricular weight: One-way ANOVA *p* = 0.000006; *, *p* < 0.05 for WIS vs. SHR, WIS vs. SHR*, and #, *p* < 0.05 SHR vs. SHR* (Student-Newman-Keuls post hoc analysis); (**B**) Right ventricular weight: One-way ANOVA: *p* ≤ 0.001; *, *p* < 0.05 for SHR* versus WIS + SHR (Student-Newman-Keuls post hoc analysis); Data are expressed as means ± S.D. and original data points are integrated. (**C**) Sirius Red Staining of left (**a**,**b**) and right (**c**,**d**) ventricular slices from SHR (**a**,**c**) and SHR* (**b**,**d**). The blue arrows mark areas with high fibrosis.

In total, seventy-six female SHRs (strain: SHR/NHsd) were analyzed and compared to normotensive rats. They were sacrificed at the ages of 2.5 months (pre-hypertensive), 7.5 months, or 11.5 months. All SHRs developed left ventricular hypertrophy in the hypertensive state. A proportion of 93% of the SHRs did not develop RVH. However 7% showed severe RVH. This finding was age-independent, and the percent of rats with RVH was 6.7% in the pre-hypertensive state, 8.3% at the age of 7.5 months, and 6.6% at the age of 11.5 months. The SHRs with severe RVH are assigned as “SHR*” in Figure 1 and in the following parts of this study and compared to SHRs without severe RVH (assigned as “SHR”) or normotensive Wistar rats (assigned as “WIS”).

### 3.2. Right Ventricular Hypertrophy in SHR/NHsd: Phenotypic Characterization

The left ventricular hypertrophy for the SHRs with established hypertension is shown in Figure 1. The means and standard deviations for left ventricular weight normalized to tibia length were 155 ± 5 mg/cm, 202 ± 17 mg/cm and 231 ± 24 mg/cm in the WIS, SHRs and SHRs*, respectively (Figure 1A). The left ventricular hypertrophy of the SHRs* exceeded that of the SHRs (Figure 1A). In contrast, the RV weight was not different between the WIS and the SHRs (32 ± 3 mg/cm and 28 ± 2 mg/cm, respectively) but strongly increased in the SHRs* (80 ± 1 mg/cm; Figure 1B; means and standard deviations for each group). A pre-hypertensive SHR* had an RV weight normalized to tibia length of 94.9 mg/cm vs. 33.4 ± 2.9 mg/cm (mean and standard deviation) in all other pre-hypertensive SHRs. The absolute values differed from adult rats, and therefore the pre-hypertensive rats were not included in Figure 1. In addition, Sirius red staining, a marker of fibrosis, indicated fibrosis in the left and right ventricles of the SHRs (Figure 1C(a,c)), but this was further enhanced in the SHRs* in both ventricles (Figure 1C(b,d)).

**Figure 2:**
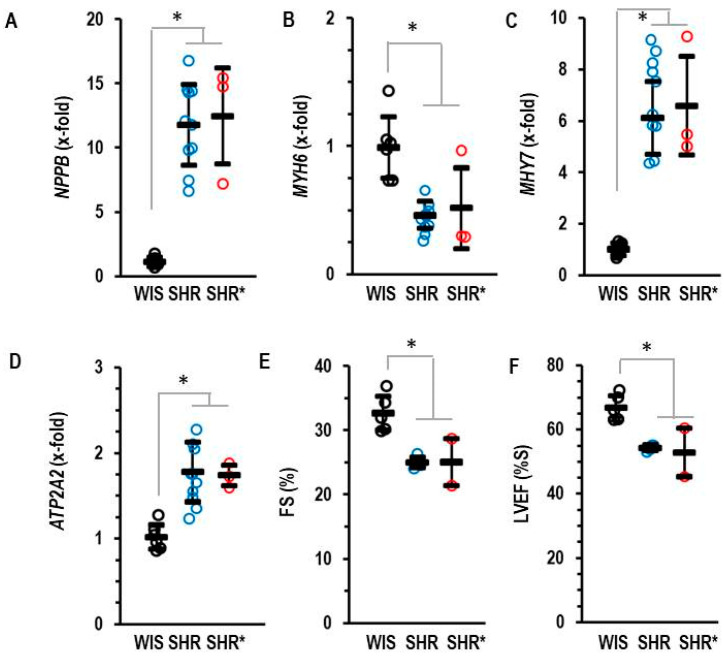
Left ventricular mRNA expression of BNP (**A**), NPPB, α-Myosin Heavy Chain (**B**) MYH6, β-Myosin Heavy Chain (**C**) MHY7 and SERCA2A (**D**) ATP2A2 analyzed in the three groups. (**E**) Fractional shortening (FS) and (**F**) left ventricular ejection fraction (LVEF) as quantified by echocardiography. Data are expressed as means ± S.D., and the original data points are integrated. One-way ANOVA: *p* ≤ 0.001; *; *p* < 0.05 vs. WIS (Student–Newman–Keuls post hoc analysis).

Compared to the normotensive rats, the SHRs and SHRs* had elevated left ventricular expression of NPPB (coding for brain natriuretic peptide, BNP), MYH7 (coding for Myosin Heavy Chain-β) and ATP2A2 (coding for SERCA2A) but reduced expression of MYH6 (coding for Myosin Heavy Chain-α) (Figure 2). Fractional shortening and the ejection fraction of the SHRs and SHRs* were slightly lower than those of the WIS (Figure 2).

In comparison to WIS, SHRs are normally lean [12,13]. This was also found in our study (Table 1). Lack of body weight gain during aging is an early disease sign in rodents. However, irrespective of RVH, the body weight did not differ between the SHRs and SHRs* (Table 1). An increased wet-weight of the liver is an independent indicator of right ventricular failure. We analyzed the wet-weights of the livers to investigate whether RVH in SHRs* is associated with symptomatic right heart failure. Liver weight was slightly increased in the SHRs* vs. SHRs (2.31 ± 0.10 g/mm vs. 2.03 ± 0.13 g/mm, respectively; Table 1; means and standard deviations).

**Figure 3:**
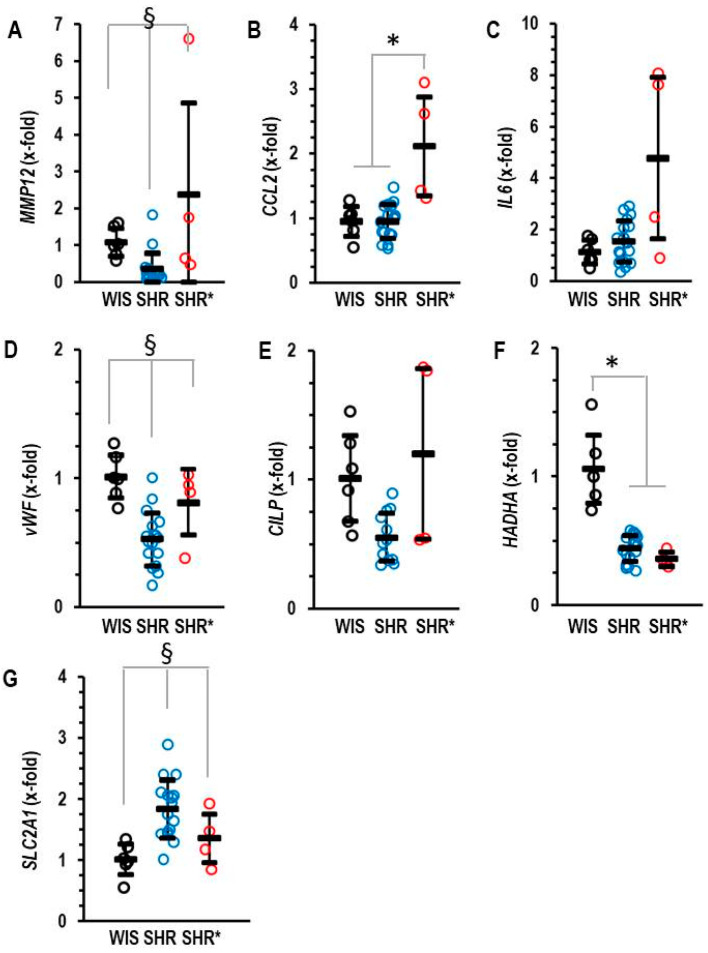
Right ventricular mRNA expression of matrix metalloprotease 12 (**A**) *MMP12*, monocyte chemotactic protein 1 (**B**) *CCL2*, interleukin 6 (**C**) *IL-6*, von Willebrand factor (**D**) *vWF*, cartilage intermediate layer protein 1 (**E**) *CILP*, trifunctional enzyme subunit α (**F**) *HADHA* and glucose transporter 1 (**G**) *SLC2A1* in the right ventricle of normotensive rats (WIS; *n* = 6), spontaneously hypertensive rats (SHRs; *n* = 17) or SHRs with right ventricular hypertrophy (SHRs*; *n* = 4). Normalization was performed to hypoxanthin-guanin-phoshoribosyltransferase (*HPRT*). A) Data are expressed as means ± S.D., and the original data points are integrated. One-way ANOVA: *p* < 0.05; *; *p* < 0.05 vs. WIS and § *p* < 0.05 vs. WIS and SHR* (Student–Newman–Keuls post hoc analysis).

The molecular signature of the RVH (SHRs*) differed from that of non-hypertrophic right ventricles (SHRs). We identified five genes that are differentially regulated between the RV from SHRs* and SHRs. These genes are *MMP12*, *CCL2*, *IL6*, *vWF* and *CILP* (Figure 3A–E). Interestingly, MMP12 is a matrix metalloprotease specific to macrophages and CCL2 is a cytokine inducing the expression of the pro-inflammatory cytokine IL-6. Similarly, vWF is linked to inflammation [14]. Therefore, the higher expression of these genes in the SHRs* vs. SHRs suggests that inflammation contributes to the onset of RVH in SHRs*. Furthermore, differences occurred in the expression of genes related to metabolism. *HADHA*, a gene coding for the trifunctional protein that belongs to the fatty acid metabolism, was down-regulated in the RV from all SHRs, but only the SHRs and not the SHRs* showed a compensatory mechanism by the up-regulation of *SLC2A1*, thereby improving glucose metabolism (Figure 3F,G).

Collectively, this type of analysis did not reveal major differences between the LV expression of the SHRs and that of the SHRs*, but there were significant differences between the RV expression of the SHRs and the SHRs*.

### 3.3. Transcriptional Alterations in the Lung from SHR with Right Ventricular Hypertrophy

**Figure 4:**
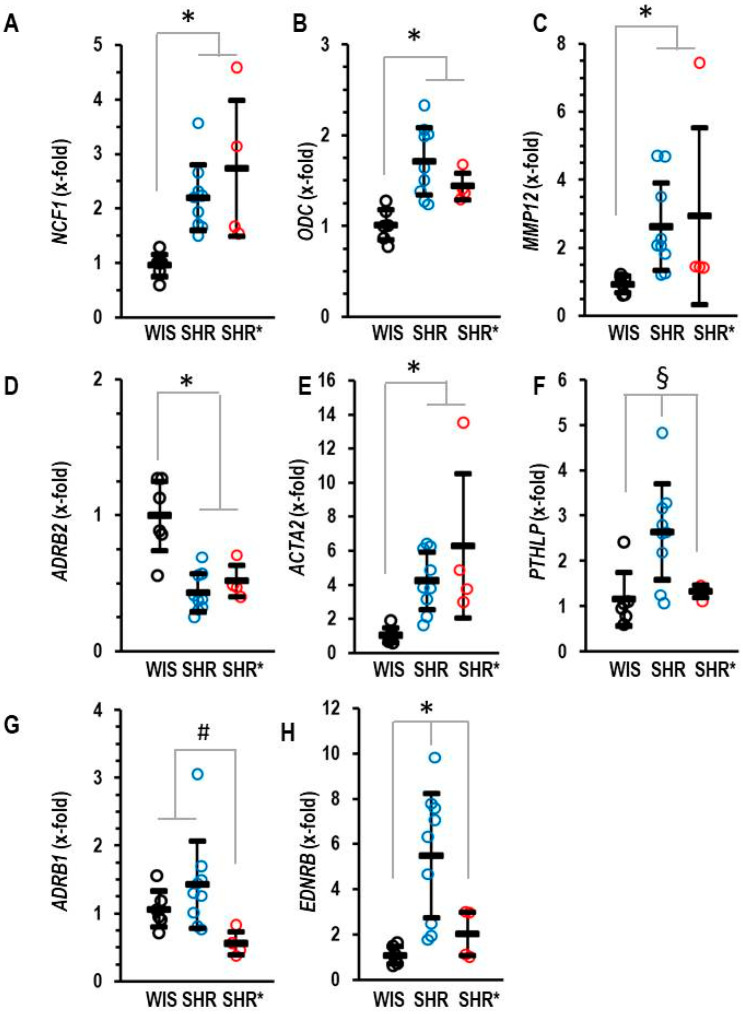
Pulmonary mRNA expression of neutrophilic cytosolic factor 1 (**A**) NCF1, ornithine decarboxylase (**B**) ODC, matrix metalloproteinase 12 (**C**) MMP12, β_2_-adrenoceptors (**D**) ADRB2, α-smooth muscle actin (**E**) ACTA2, parathyroid hormone-related peptide (**F**) PTHLP, β_2_-adrenoceptors (**G**) ADRB1 and endothelin receptor type B (**H**) EDNRB of normotensive rats (WIS; *n* = 6), spontaneously hypertensive rats (SHRs; *n* = 9) or SHRs with right ventricular hypertrophy (SHR*; *n* = 4). Normalization was performed to β_2_-microglobulin (B2M). Data are expressed as means ± S.D., and the original data points are integrated. ANOVA: *p* < 0.05; *; *p* < 0.05 vs. WIS; #, *p* < 0.05 vs. SHR*; §, *p* < 0.05 vs. SHR (Student–Newman–Keuls post hoc analysis).

We hypothesized that the differences in pulmonary gene expression between the SHRs and the SHRs* may give us a hint as to how to explain RVH in SHR*. In comparison to WIS, SHRs showed an increased expression of *NCF1*, *ODC*, *MMP12* and *ACTA2*, as well as a decreased expression of *ADRB2* (Figure 4A–E). However, all these genes were not differentially expressed between SHRs and SHRs*. Therefore, the altered expression in the SHRs versus the WIS cannot explain the different phenotypes between the SHRs and the SHRs*. In contrast, three genes were an exception from this common regulation. These are *PTHLP*, *EDNRB* and *ADRB1*, which are expressed at higher levels in SHRs compared to SHRs* (Figure 4F–H).

### 3.4. Effect of Low Endothelin B Receptor Expression on Pulmonary Concentration of Endothelin-1

**Figure 5:**
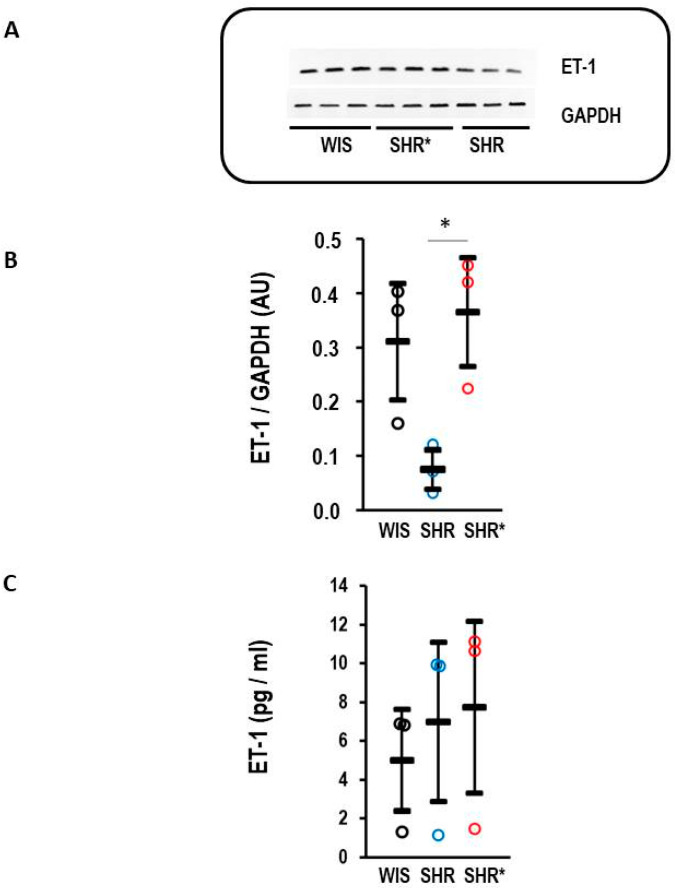
Endothelin-1 (ET-1) content in the lung and plasma. (**A**) Representative Western blot with samples from WIS, SHRs* and SHRs. (**B**) Quantification of the Western blot shown in A with normalization to GAPDH as loading control. Kruskal–Wallis test: *p* = 0.014; *, *p*< 0.05 for SHRs vs. SHRs*. (**C**) Plasma concentration of endothelin-1 for normotensive rats (WIS, *n* = 6), spontaneously hypertensive rats (SHRs, *n* = 9) and SHRs with right ventricular hypertrophy (SHRs*; *n* = 4); one-way ANOVA: *p* = 0.197. Data are expressed as means ± S.D., and the original data points are integrated.

The endothelin receptor type B is a clearance receptor for endothelin-1 [15]. Therefore, it was investigated whether the observed high expression of EDNRB, the gene coding for the endothelin receptor type B, affects the concentration of endothelin-1 in the lungs. Indeed, the SHRs with a high expression of EDNRB had a lower endothelin-1 protein content compared to the WIS or SHRs* (Figure 5A,B). This regulation is organ-specific, as plasma concentrations of endothelin-1 remained similar between the three strains (Figure 5C).

**Figure 6:**
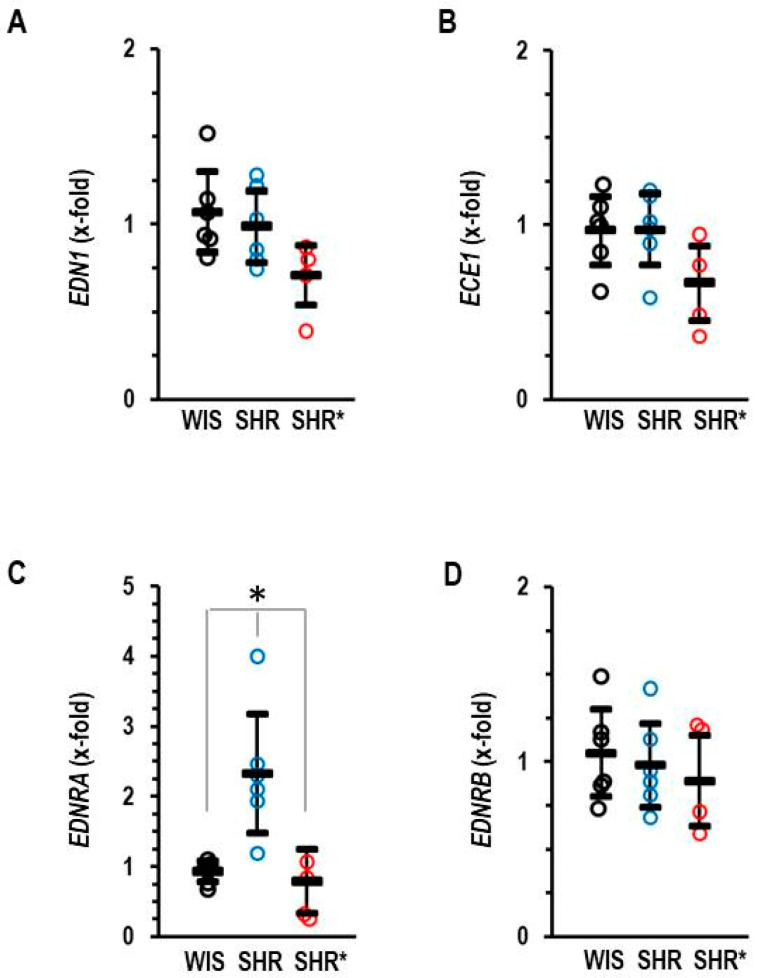
Right ventricular mRNA expression of endothelin-1 (**A**) EDN1, endothelin-converting enzyme (**B**) ECE1, endothelin receptor type A (**C**) EDNRA and endothelin receptor type B (**D**) EDNRB of normotensive rats (WIS; *n* = 6), spontaneously hypertensive rats (SHRs; *n* = 6) or SHRs with right ventricular hypertrophy (SHRs*; *n* = 4). EDNRB: one-way ANOVA: *p* = 0.796; EDNRA: one-way ANOVA: *p* = 0.001; *; *p* < 0.05 SHRs vs. WIS + SHRs*; ECE1: one-way ANOVA: *p* = 0.081; END1: one-way ANOVA: *p* = 0.070. Data are expressed as means ± S.D., and the original data points are integrated.

In addition, we analyzed the RV expression of the endothelin systems, as this was differentially regulated in the lungs. In the RV, our most important observation is that SHRs have a higher expression of the EDNRA compared to SHRs*, whereas the mRNA expression of endothlin-1, endothelin-converting enzyme and endothelin receptor type B remained unaffected (Figure 6A–D).

**Figure 7:**
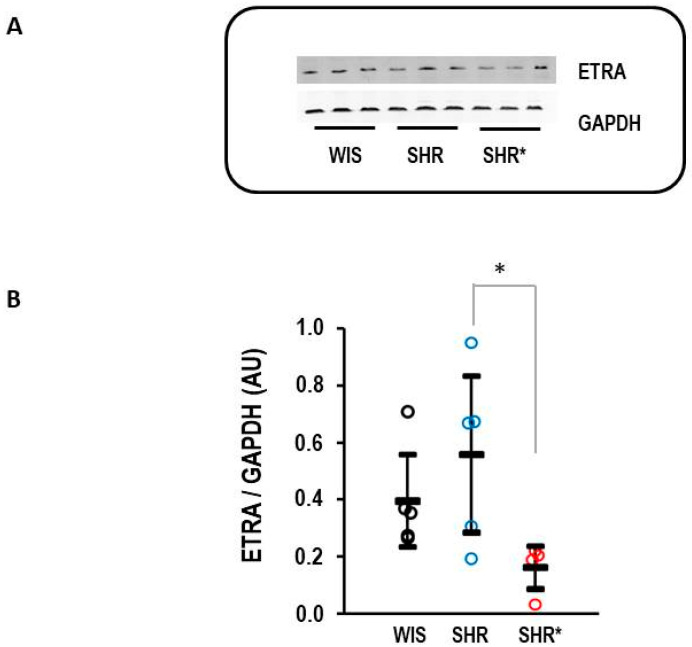
Right ventricular protein expression of endothelin receptor type A (ETRA). (**A**) Representative Western blot. (**B**) Quantification of protein expression. Data are given for normotensive rats (WIS; *n* = 5), spontaneously hypertensive rats (SHRs, *n* = 5), and SHRs with right ventricular hypertrophy (*n* = 4). Kruskal–Wallis test: *p* = 0.038; *, *p* = 0.021 for SHR vs. SHR* (least significant difference test). Data are expressed as means ± S.D., and the original data points are integrated.

Finally, the difference in the expression of EDNRA between the SHRs and SHRs* could be confirmed at the protein level (Figure 7).

## 4. Discussion

SHRs develop systemic hypertension but mostly mild or no pulmonary hypertension and no RVH. However, there are a few exceptions reported in the literature. We analyzed female rats from the SHR/NHsd strain, and the results were mainly in agreement with these assumptions. However, a sub-group of rats from the SHR/NHsd strain developed severe RVH irrespective of the age of the animals. In total, a rate of 7% for the SHRs* was remarkably constant throughout aging. The rats that were identified as rats with RVH when finally sacrificed (assigned here as SHRs*) did not differ in routine scoring from the SHRs without RVH when they were alive. This suggests that a sub-group of the strain displays a genetic variance that is associated with severe RVH but mild RV dysfunction. Indeed, the phenotype of the SHRs* was mild: the rats were not conspicuous during health scoring, had normal weight gain, and liver wet weight was not strongly different from SHRs. This study addressed two questions. First, can transcriptional modifications in pulmonary tissue explain RVH in SHRs*? Second, can molecular adaptations in the RV explain the mild phenotype of RVH in SHRs*? The main finding of our study is that in the lungs of SHRs*, the endothelin clearing receptor (endothelin receptor type B) is down-regulated, leading to an increase in the pulmonary endothelin-1 concentration. Endothelin is a very powerful vasoconstrictor, and targeting the pulmonary endothelin system is an approved therapy for this indication [16]. Therefore, our data suggest that the observed increase in endothelin-1 concentration contributes to the phenotype of SHRs*. In addition, the increased expression of *ACTA2* in the lungs indicates vascular remodeling in the pulmonary circulation that favors pulmonary hypertension as well [17]. Although we could not directly prove whether SHRs* have pulmonary hypertension, increased endothelin-1 and *ACTA2* expression predicts an increase in RV afterload and subsequent RVH. This is exactly the phenotype we observed.

The inhibition of endothelin type 1 receptors is one of the best accepted therapies in pulmonary hypertension, and reducing endothelin-1 activity reduces RVH in rats [17,18]. Endothelin-1 is a strong vasoconstrictor and induces smooth muscle cell proliferation. The endothelin-1 effect on smooth muscle cells is mediated via endothelin type A and type B receptors [19]. However, type B endothelin receptors are also expressed on endothelial cells. These cells are the main source of endothelin-1. In endothelial cells, type B receptors act as clearing receptors for endothelin-1, lowering the concentration of endothelin-1 in their surroundings. We found that SHRs express a large amount of *EDNRB* compared to normotensive rats. Similarly, these rats had the lowest local concentration of endothelin-1. This suggests that transcriptional up-regulation of endothelin receptors type B is a compensatory mechanism of SHRs to avoid pulmonary hypertension. However, in the SHRs*, this up-regulation was much smaller, and the local concentration of endothelin-1 was higher than in the SHRs. The data suggest that SHRs are very sensitive to endothelin-1 but that the compensatory up-regulation of type B receptors normally prevents pulmonary hypertension. Such a clearing function in the pulmonary circulation was proven before [20,21,22]. In line with these assumptions, the genetic deficiency of endothelin B receptor expression potentiates endothelin-1 vasoconstriction and induces pulmonary hypertension [23]. Similarly, experimental pulmonary hypertension induced in lambs down-regulates the expression of endothelin type B receptors [24,25]. Alternatively, SHRs* may have an excessive type of left ventricular hypertrophy triggering pulmonary modifications secondary to excessive left heart failure, as in SHR-SP [26]. Indeed, the SHRs* had stronger left ventricular hypertrophy compared to the SHRs. However, neither the molecular adaptation to pressure overload nor the left ventricular function differed between SHRs and SHRs*. This makes it unlikely that excessive left ventricular hypertrophy triggers the observed changes in endothelin-1 and *ACTA2* in the lungs. It was postulated before that SHRs are more sensitive to endothelin-1 compared to normotensive rats [27]. In contrast to changes in the pulmonary concentration of endothelin-1, the systemic concentration of endothelin-1 did not differ between SHRs and SHRs*. Furthermore, there was no general up-regulation of *EDNRB* in SHRs, as for example, its expression in the RV was not differentially regulated in the same rats. Such findings support the idea that the up-regulation of *EDNRB* in SHRs is specific for the lungs. In conclusion, we identified differences in the pulmonary expression of genes linked to pulmonary hypertension in a sub-group of these rats that can explain the large heterogeneity described for pulmonary pressure in these rats.

Given the severe RVH in SHRs*, exceeding the RV weight of SHRs by a factor of 2.5, it is surprisingly that this type of hypertrophy had relatively mild functional consequences.

The RV of the SHRs* had clear signs of inflammation compared to the non-hypertrophic RV of the SHRs. This is indicated by an increased expression of *MMP12*, *CCL2*, *IL-6* and *vWF*. It would be interesting to address the question whether inflammation triggers hypertrophic growth directly. Nevertheless, the effect on RV function remained moderate. An explanation for this observation may come from the finding that these RVs express less endothelin type A receptors. Stimulation of endothelin receptors type A does not necessarily indicate mal-adaptive remodeling [28]. However, in SHRs the response of cardiomyocytes to endothelin-1 may be different [29]. Therefore, the stimulation of endothelin-1 type A receptors may trigger mal-adaptive hypertrophy in the RV. Down-regulation of endothelin-1 type A receptors in the RV, as seen here in the SHRs*, would then be an adaptive mechanism. Similarly, the pharmacological inhibition of endothelin type 1 receptors shows improved function and exercise tolerance in patients with pulmonary hypertension and RVH [11]. In addition, the SHRs* had the lowest expression of *END1* and *ECE1*. This may add to the low endothelin signaling in the right ventricle of the SHRs*. Finally, the differential expression of *CILP* in the RV is in line with the finding that CILP is a reasonable biomarker for RVH [30].

Our experimental observation of bi-ventricular hypertrophy in the context of systemic hypertension in some but not all SHRs is in line with clinical observations showing bi-ventricular hypertrophy in hypertensive patients of approximately 17–29% of all cases [31,32]. As essential hypertension is a monogenic disease in rodents and humans, it is important to identify the genes that trigger such complications. Furthermore, our study gives a rational explanation to the divergence in former reports about the occurrence of pulmonary hypertension in SHRs and may help to understand the different outcomes in studies dealing with different SHR strains. Therefore, it is important to precisely describe the strains used in experimental studies. A lack of reproducibility of data in animal experiments may reflect inter-individual heterogeneity, as shown here within one strain rather than an intrinsic problem with animal experiments.

## 5. Conclusions

Our observation of phenotypic variation in female SHR/NHsd rats leading to severe RVH allows for a better understanding of the requirements for RVH and right heart failure. Our observation, that SHRs* have a severe RVH but a moderate phenotype in the presence of low RV expression of endothelin A receptors, highlights the importance of this pathway for the transition of RVH to right heart failure. The constant rate of approximately 7% of such rats in this strain during aging suggests a genetic basis for this phenotype.

## Figures and Tables

**Table 1:** Body, lung and atrial weights of rat strains.

	BW/TL (g/cm)	LW/TL (g/cm)	LAW/TL (mg/cm)
WIS (*n* = 6)	81.18 ± 3.50	2.16 ± 0.13	10.51 ± 1.59
SHR (*n* = 10)	60.37 ± 3.82 *	2.03 ± 0.13 ^#^	16.40 ± 4.82 ^$^
SHR* (*n* = 4)	65.65 ± 5.43	2.31 ± 0.23	10.31 ± 3.69

Data are means ± S.D.; *; *p* = 0.001 vs. WIS (Kruskal–Wallis test); ^#^, *p* = 0.040 vs. WIS (ANOVA with Student–Newman–Keuls post hoc analysis); ^$^, *p* = 0.035 vs. SHRs* and *p* = 0.017 vs. WIS; ANOVA with least significant difference test; BW = body weight; TL = tibia length; LW = liver weight; LAW = left atrial weight.

## Data Availability

The raw data and subsequent analysis of the data are stored at the Max-Planck Institute Bad Nauheim and will be shared by the lead contact on request.

## Supplementary Materials

The following supporting information can be downloaded at: https://www.mdpi.com/article/10.3390/biology13100752/s1, Table S1: List of primers used in this study.
