# Supplementary material for: Right Ventricular Hypertrophy in Spontaneously Hypertensive Rats (SHR/NHsd) Is Associated with Inter-Individual Variations of the Pulmonary Endothelin System
Source: Biology (Basel). 2024 Sep 24;13(10):752. doi: 10.3390/biology13100752 (PMC11505455; doi:10.3390/biology13100752)
Supplement: Supplementary file 1 [file biology-13-00752-s001.zip › biology-3180627-supplementary.pdf]

Supplement Table S1: List of primers used in this study

	Forward	Reverse
ACTA2	ACCATCGGGAATGAACGCTT	CTGTCAGCAATGCCTGGGTA
ACTC1	ACCACCGCTGAACGTGAAAT	AGTCTCAGGACAGCGGAAAC
ACTN2	AGTGCCAGCTGGAGATCAAC	AGCGCTCCAGTCTTCGAATC
ADRB1	GGCGCTCATCGTGCTGCTCA	AGGCACCACCAGCAGTCCCA
ADRB2	GCTTCTGTGCCTTCGCCGGT	AGCCTTCCATGCCAGGGGCT
ARG1	GGAAGCATCTCTGGCCACGCC	CACCGGTTGCCCGTGCAGAT
ARG2	TGAGGAGCAGCGTCTCCCGT	GCTTCTCGGATGGCGGCTGG
B2M	GCCGTCGTGCTTGCCATTC	CTGAGGTGGGTGGAACTGAGAC
CCL2	TCACGCTTCTGGGCCTGTTGT	TCCAGCCGACTCATTGGGATCA
CDH5	CCAGAATTTGCCCAGCCCTA	GTCCTCGTTCTTCAGGGCAA
COL1A1	GCGAACAAGGTGACAGAG	CCAGGAGAACCAGCAGAG
COL3A1	TGGAGTCGGAGGAATG	GCCAGATGGACCAATAG
CYBA	CCTCCACTTACTGCTGTCCG	GTAGGTGGCTGCTTGATGGT
CYBB	GTTTGCCGGAAACCCTCCTA	CCTTCTGCTGAGATCGCCAA
CXCL12	CCAAGGTCGTCGCCGTGCTG	GGCTCTGGCGACATGGCTCT
CXCR4	GCCATGGCTGACTGGTACTT	CACCCACATAGACGGCCTTT
DES	GCGCTTTTTGGAACAGCAGA	GCCTCTGCAGGTCGTCTATC
DNM1L	TGGAAAGAGCTCAGTGCTGG	TCAACTCCATTTTCTTCTCCTGT
ECE1	TCTGGCCAACATCACCATCC	TAGACCACGATGGGCTCAGA
EDN1	CCGTATGGACTAGGAAGCCC	TGCATGGTACTTTGGGCTCG
EDNRA	ATTTGGCCCTGCCTAGCAAT	CCCACCATTCCCACGATGAA
EDNRB	GCTAGCCATCACTGCGATCT	TGTCTTGGCCACTTCTCGTC
EFHD2	AACTTCTTCGAGGCCAAGGT	TTAAAGGCCGCTTTTCGCTG
ELN	GCCCTGGTGTTGGTGGCGTT	GCCGGTGCCAGGACCAGTTC
FGF2	TCCATCAAGGGAGTGTGTGC	TCCGTGACCGGTAAGTGTTG
HADHA	ACATCGGAGCAGTCTTTGGG	AGCTGACACGGGGTAAACTG
IL6	CACTTCACAAGTCGGAGGCT	TCTGACAGTGCATCATCGCT
IL6R	TGGTCAAAGACGTTCACGGT	CTCTGCTAGCCAAGGAGTGC
LOX	ACAACCGCACTGCCTCTGCC	GCCTTGAGGCTCCATCGCCG
MEF2C	CAGTTGGGAGACCGTACCAC	GTGAGTCCAATGGGGGAGTG
MMP2	ACAACAGCTGTACCACCGAG	GGACATAGCAGTCTCTGGGC
MMP9	CAATCCTTGCAATGTGGATG	AAATCTTCTTGGACTGCGGA
MMP12	TGCAGCTGTCTTTGATCCAC	GCATCAATTTTTGGCCTGAT
MYH6	GCTGGGGCTTCAGAAGACAT	CCTCCTCAGGACTTTTTCGGT
MYH7	TACAGGTGCATCAGCTCCAG	TGGCACCGTGGACTACAATA
NCF1	GCACCACCTCGCAGGTCGAC	GCGCTGCTGCAGGAATCGGA
NCF2	ATGGTGCCAACAGCTCGGCC	GCTGGCTGTAGGGGAGGCCA
NFE2L2	TTGTAGATGACCATGAGTCGC	GCCAAACTTGCTCCATGTCC
NOS2	AAGAGACGCACAGGCAGAG	CAGCAGGCACACGCAATG
NOS3	CAACTGGAAAAAGGCAGCCC	AAGAGCCTCTAGCTCCTGCT
NPPA	ATGGGCTCCTTCTCCATCAC	TCTTCGGTACCGGAAGCTG
NPPB	ATGATTCTGCTCCTGCTTTTCC	TCTGCATCGTGGATTGTTCTG
ODC1	GAAGATGAGTCAAACGAGCA	AGTAGATGTTTGGCCTCTGG
OSM	ACACTGCTTAGTTTGGCCCT	TCGCCTGACTCTTCAACTGG
OSMR	TAGTCATTCTGGACATGAAGAGGT	CGTTGGGTCTGAGAATGTGC
PCSK9	TTGAACAAACTGCCCATCGC	CCCAACAGGTCACTGCTCAT
PLIN2	GCCCGAGTCACAACCCCACG	AGAGTCGACAGCCGCTCGGT
PPARGC1A	AGTGCTCAGCCGAGGACACGA	TGCCCCTGCCAGTCACAGGA
PPARG	TCGCTGATGCACTGCCTATG	CACAATCCCCTCCTGCAACT
PTH1R	GTGAGGTGCAGGCAGAGATT	TTTTGGTGTTGGGAGCAGGT
PTHLH	AGCTACTCCGTGCCCTCCCG	AGGAAGAAACGGCGGCGCAA
SLC2A1	GCTGTGGCTGGCTTCTCTAA	CCGGAAGCGATCTCATCGAA
SLC2A4	ACCGTCTTCACGTTGGTCTC	ATCAAGATGGCACAGCCACA

SOD1	AAGGCCGTGTGCGTGCTGAA	ACATGGAACCCATGCTCGCCT
SOD2	ATGTTGTGTCGGGCGGCGTG	TCGCGTGGTGCTTGCTGTGG
SOD3	TGCTGCCTCCCGATCAGCCA	CCCTGGCTCAGGTCCCCGAA
SST	CCCAACCAGACAGAGAACGA	AAGTTCTTGCAGCCAGCTTTG
TGFB1	ATTCCTGGCGTTACCTTGG	CCTGTATTCCGTCTCCTTGG
UCP2	ATCTTCTCAGCCGGCGTTTC	AGGGTGGTGATGGTCCCTAA
UCP3	GATCTCCTCACCTTCCCCCT	AGGCAAAACTCATCTGGCGA
VEGFA	TGCCCCTAATGCGGTGTGCG	GGCTCACAGTGAACGCTCCAGG
vWF	AAGATGGCAAGAGAGTGGGC	CCGTAGGCCTCACTGGAAAG
